# Special Issue: “Potential Prevention and Treatment of Neurodegenerative Disorders”

**DOI:** 10.3390/ijms27167458

**Published:** 2026-08-20

**Authors:** Maria Paola Bertuccio

**Affiliations:** Department of Biomedical and Dental Sciences and Morpho-Functional Imaging, University of Messina, 98125 Messina, Italy; mbertuccio@unime.it

The continuous increase in life expectancy has led to a growing global burden of neurodegenerative disorders. These conditions are characterized by the progressive deterioration of neuronal structure and function, ultimately resulting in neuronal loss within different regions of the nervous system [1]. Despite their distinct etiologies and clinical manifestations, the most prevalent neurodegenerative diseases as Alzheimer’s disease (AD), Parkinson’s disease (PD), multiple sclerosis (MS) and amyotrophic lateral sclerosis (ALS), share a common feature of progressive neuronal dysfunction and loss, which can lead to cognitive, motor, and other neurological impairments, substantially affecting patients’ quality of life [2]. Unfortunately, the mechanisms underlying the loss of bodily functions have not yet been fully elucidated, also due to the complexity of the etiology and pathogenesis of various neurodegenerative disorders. Growing evidence indicates that abnormal protein aggregation, oxidative stress, mitochondrial dysfunction, neuroinflammation, and impaired neurotrophic signaling represent major contributors to the pathogenesis of neurodegenerative diseases [3,4,5]. In recent years, natural bioactive compounds have gained increasing attention as promising multi-target therapeutic candidates owing to their antioxidant, anti-aggregative, and anti-apoptotic activities [5,6]. By targeting multiple pathogenic processes simultaneously, these compounds may serve as valuable adjunctive agents to conventional therapies, potentially enhancing neuroprotection and slowing disease progression. In addition to natural bioactive compounds, emerging therapeutic strategies such as synthetic drugs and personalized medicine are being explored to counteract oxidative stress-related neurological disorders [7]. However, current pharmacological interventions for neurodegenerative diseases remain largely limited to symptomatic management, as disease-modifying therapies have yet to be established. Consequently, there is an urgent need to develop novel preventive and therapeutic strategies aimed at delaying neurodegenerative disorders onset, slowing their progression, or ultimately achieving effective treatment.

This Special Issue titled “Potential Prevention and Treatment of Neurodegenerative Disorders” of the International Journal of Molecular Sciences consists of two original research articles and three review articles that provide insights into innovative treatment options and novel biomarkers as potential therapeutic targets for the treatment of neurodegenerative diseases.

The article authored by Stefania Merighi and colleagues (Contribution 1) explored the potential neuroprotective effects of pulsed electromagnetic fields (PEMFs) in PC12 cells, a cellular model of AD [8], exposed to hydrogen peroxide (H_2_O_2_) or amyloid-β peptide (Aβ), causing an exacerbation of oxidative damage. Through the assessment of cell viability, mitochondrial function, oxidative stress, and the activation of key signaling pathways, this in vitro study attempts to elucidate the mechanisms underlying the neuroprotective effects of PEMFs and their role in promoting neuronal resilience. PEMFs seem to play a neuroprotective role by modulating multiple signaling pathways and may offer a non-invasive and potentially safer alternative to current drug treatments for Alzheimer’s disease.

Several natural products also possess neuroprotective effects representing promising curative capabilities for neurodegenerative diseases. Xiaoyu Du and colleagues (Contribution 2) investigated the properties of rutin, a natural flavonoid glycoside, in a superoxide dismutase 1 (SOD1)-G93A mouse model of ALS. Rutin possesses a wide range of biological activities, including antioxidant, anti-inflammatory, and antiapoptotic effects, to name a few, and, thanks to its ability to cross the blood–brain barrier, exerts beneficial effects on the central nervous system, such as enhancing neuronal signal transduction and improving learning and memory [9]. It is well known that genetic mutations in the SOD1 gene are associated with familial ALS [10] and the authors’ findings highlighted the ability of rutin to alleviate motor deficits, mitigate motor neuron degeneration and reduce neuroinflammation in SOD1-G93A mice, supporting its potential therapeutic value in ALS.

Another potential therapeutic target in neurodegenerative diseases is 5-hydroxytryptamine, or serotonin (5-HT), a key neurotransmitter involved in the regulation of several physiological processes [11]. Cencan Xing and colleagues (Contribution 3) summarized the current evidence on the role of 5-HT in neurodegenerative disorders, focusing on 5-HT receptors and their function in AD, PD, ALS, MS and Huntington disease (HD). 5-Hydroxytryptamine (5-HT) exerts a wide range of neuroprotective effects by promoting neuronal survival and maintaining neuronal function. Beyond its direct actions on the nervous system, 5-HT displays anti-inflammatory and immunomodulatory properties, regulating immune cell activity and modulating the magnitude of inflammatory responses. Through the activation of specific receptor subtypes, 5-HT plays a pivotal role in the modulation of neurotransmission, a mechanism that is particularly relevant in the pathophysiology of neurodegenerative diseases. In addition, 5-HT is critically involved in neuronal differentiation, migration, and synaptogenesis, thereby contributing to the development, organization, and functional maintenance of neural circuits.

Alzheimer’s disease represents the most common cause of dementia and is strongly associated with sleep disturbances [12]. Therefore, it is of interest to investigate the effects of drugs on this symptom. Specifically, in their systematic review, Luis Fernando Chavez-Mendoza and colleagues (Contribution 4) analyzed the effect of benzodiazepines and Z-drugs on this disorder. Indeed, the early diagnosis and treatment of sleep disorders may represent an important strategy for reducing the risk of cognitive decline, delaying its progression to dementia, and limiting the deterioration of pre-existing cognitive impairment. This systematic review highlights the greater ability of Z-drugs compared to benzodiazepines to improve sleep architecture and dementia-related symptoms, such as memory and language skills.

A common aspect of various neurodegenerative diseases is endothelial dysfunction, which may be linked to the alteration in pathways involving nitric oxide (NO) production that are, in turn, associated with hyperhomocysteinemia (HHcy) and elevated asymmetric dimethylarginine (ADMA) concentrations [13]. In their narrative review, Caterina Saija and colleagues (Contribution 5) examined the interplay among HHcy, ADMA, and vitamins in PD, AD and MS, highlighting the dysfunctional pathways and neuropathological processes involved. The review further provides a comprehensive overview of the potential effects of vitamin supplementation in these neurodegenerative diseases, highlighting its potential role in modulating oxidative stress and inflammatory processes and in restoring physiological Hcy metabolism.

I wish to express my sincere gratitude to all authors, reviewers, and editorial board members for their invaluable contributions to this Special Issue. I hope that the articles presented in this collection will offer meaningful insights, stimulate interdisciplinary collaboration, and inspire further research on integrated diagnostic approaches and personalized therapeutic strategies, improving the clinical management of neurodegenerative diseases and the quality of life of patients and their families.
